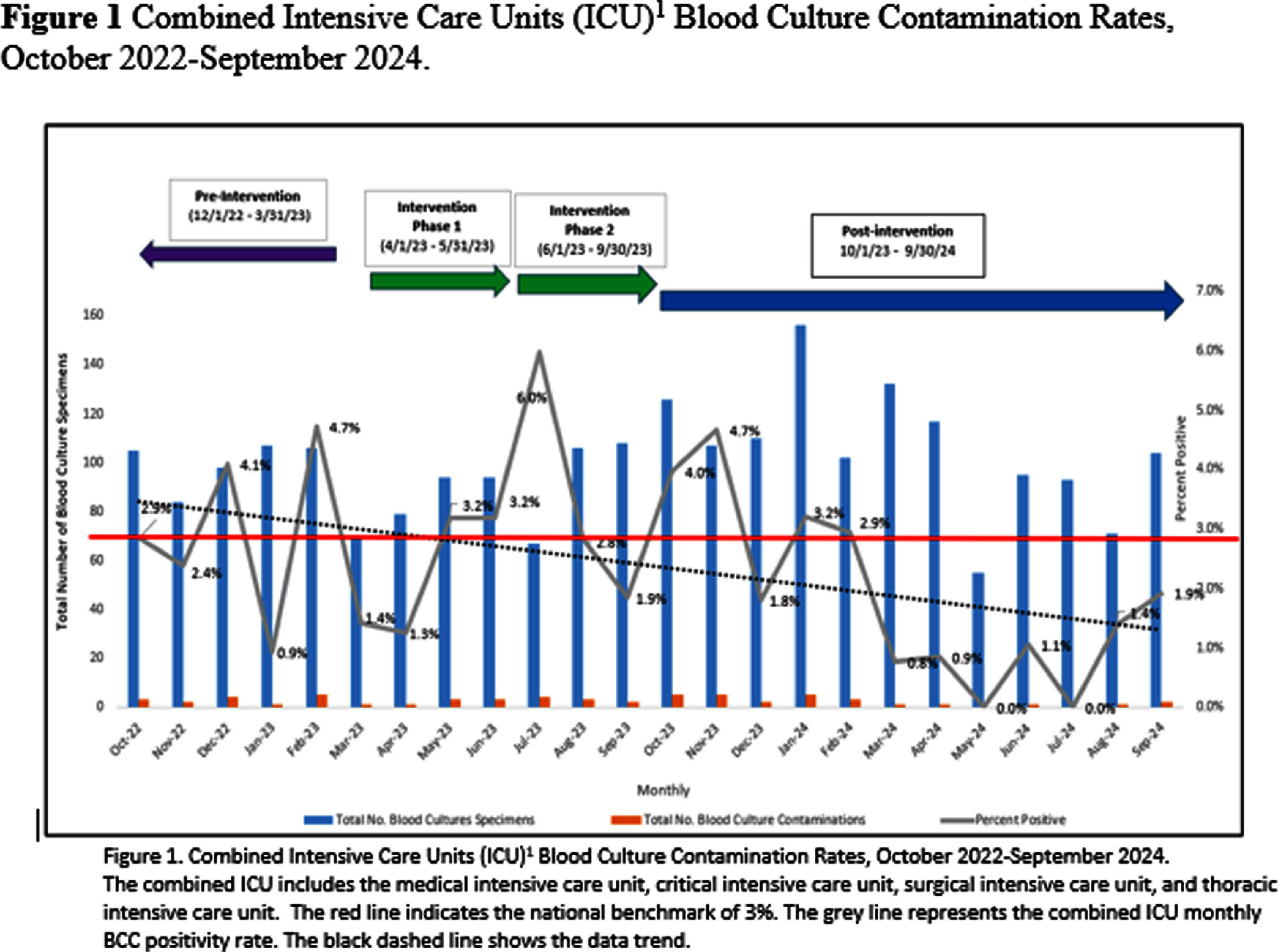# Measures implemented to reduce Blood Culture Contamination in Intensive Care Units at a Veteran’s Administration Hospital

**DOI:** 10.1017/ash.2025.393

**Published:** 2025-09-24

**Authors:** Aderonke Badejogbin, Sherry R. Reid, Ikwo Oboho, Denisse Silva, Tanaya Lindstrom, Mary Ramirez, Cinemol Varghese, Moses Njeri, Emily Mogeni, Patricia Shahim

**Affiliations:** 1 VA North Texas Health Care System; 2 Veterans Affairs North Texas Health Care System; 3 VA North Texas Health Care System/UT Southwestern University Medical Center; 4 VA North Texas Health Care System, Dallas, Texas; 5 VA Medical Center Dallas; 6 VA North Texas Healthcare System; 7 VA Medical Center; 8 Dallas Veteran Affairs Medical Center

## Abstract

**Background:** Blood Culture Contamination (BCC) is a significant safety and quality indicator for intensive care units (ICU) at the Veteran Affairs North Texas Healthcare System. In February 2023, the combined ICU BCC rate was 4.7%. The American Society for Microbiology and the Clinical Laboratory Standards Institute recommends a BCC rate not exceed 3%. **Methods:** In March 2023, a multidisciplinary workgroup was created to reduce the combined ICU BCC rate to a target goal of evidence-based standardized process was implemented using a blood culture kit and guide, hand hygiene, site prep, and aseptic technique. Nurses were also educated to avoid drawing from existing lines. In phase two, a second verifier was added to observe blood culture draws, and documentation fields were modified to record the verifier’s name and location. Training reinforced hand hygiene, use of clean gloves, site prep, and cleaning bottle tops with alcohol. In addition, the Microbiology supervisor disseminated monthly BCC reports to key stakeholders. BCC Champions used reports to monitor compliance with processes, and if deficits were detected, feedback was provided to nurses for immediate corrective action. **Results:** In the 6-month pre-intervention period (12/1/22 – 3/31/23), 16 BCC events occurred from 570 blood cultures, 2.7% BCC rate. In the 6-month intervention period (4/1/23 – 9/30/23), 16 BCC events occurred from 548 blood cultures collected, 3.1% BCC rate (Phase 1: 2.2% BCC rate, Phase 2, 3.5% BCC rate). The BCC rate reduced by 60% from a peak of 4.7% in the pre-intervention period (2/23) to 1.9% (9/23). In May 2024, a new blood culture kit was piloted and adopted for use in the ICU. Quarterly workgroup meetings were implemented to monitor the quality initiative. In the 12-months post-intervention (10/23 – 9/24), the ICU BCC rate was 1.9%. **Conclusion:** We reduced the ICU BCC rate to Reducing BCC may lower healthcare costs and reduce unnecessary antibiotic use.

**Figure:**